# Whole lung lavage therapy for pulmonary alveolar proteinosis: a global survey of current practices and procedures

**DOI:** 10.1186/s13023-016-0497-9

**Published:** 2016-08-31

**Authors:** Ilaria Campo, Maurizio Luisetti, Matthias Griese, Bruce C. Trapnell, Francesco Bonella, Jan Grutters, Koh Nakata, Coline H. M. Van Moorsel, Ulrich Costabel, Vincent Cottin, Toshio Ichiwata, Yoshikazu Inoue, Antonio Braschi, Giacomo Bonizzoni, Giorgio A. Iotti, Carmine Tinelli, Giuseppe Rodi, for the WLL International Study Group, Toru Arai, Andrey A. Bazhanov, Issahar Ben-Dov, Francesco Bonella, Antonio Braschi, Alicia Casey, Ulrich Costabel, Vincent Cottin, Deniz Dogru, Wolfgang Gesierich, Matthias Griese, Jan C. Grutters, Maija Halme, Michael Henry, Felix J. F. Herth, Wang Hui-ying, Toshio Ichiwata, Julia M. Ilkovich, Yoshikazu Inoue, Giorgio A. Iotti, Sarosh Irani, Vítězslav Kolek, António Morais, Cliff Morgan, Thomas Nicolai, Lubov N. Novikova, Robert Primhak, Karl Reiter, George Retsch-Bogart, Giuseppe Rodi, Eric Russi, Jochen Schmitz, Carola Schön, Christian Schumann, Marcel Veltkamp, Charl Verwey, Robert E. Wood

**Affiliations:** 1 Pneumology Unit, Fondazione IRCCS Policlinico San Matteo and University of Pavia, Pavia, Italy; 2 Kinderklinik und Kinderpoliklinik im Dr. von Haunerschen Kinderspital, University of Munich, Munich, Germany; 3 Translational Pulmonary Science Centre, Cincinnati Children’s Hospital, Cincinnati, OH USA; 4 Interstitial and Rare Lung Disease Unit, Ruhrlandklinik University Hospital, University of Duisburg-Essen, Essen, Germany; 5 Centre of Interstitial Lung Diseases, St. Antonius Hospital Nieuwegein, Nieuwegein, The Netherlands; 6 Niigata University Medical and Dental School, Niigata, Japan; 7 National Reference Centre for Rare Pulmonary Disease, Hopital Louis Pradel, Lyon, France; 8 Tokyo Medical University Hachioji Medical Center, Tokyo, Japan; 9 Department of Diffuse Lung Diseases and Respiratory Failure, Clinical Research Centre, National Hospital Organization Kinki-Chuo Chest Medical Centre, Osaka, Japan; 10 Department of Anesthesiology and Intensive Care, Fondazione IRCCS Policlinico San Matteo, University of Pavia, Pavia, Italy; 11 Clinical Epidemiology and Biometric Unit, Fondazione IRCCS Policlinico San Matteo, Pavia, Italy; 12 Laboratorio di Biochimica e Genetica, S.C. Pneumologia, Fondazione IRCCS Policlinico San Matteo, via Taramelli 5, 27100 Pavia, Italy

**Keywords:** Pulmonary alveolar proteinosis, Whole lung lavage, Rare disease, Interstitial lung disease

## Abstract

**Background:**

Whole lung lavage (WLL) is the current standard of care treatment for patients affected by pulmonary alveolar proteinosis (PAP). However, WLL is not standardized and international consensus documents are lacking.

Our aim was to obtain a factual portrayal of WLL as currently practiced with respect to the procedure, indications for its use, evaluation of therapeutic benefit and complication rate.

**Methods:**

A clinical practice survey was conducted globally by means of a questionnaire and included 27 centers performing WLL in pediatric and/or adult PAP patients.

**Results:**

We collected completed questionnaires from 20 centres in 14 countries, practicing WLL in adults and 10 centers in 6 countries, practicing WLL in pediatric patients.

WLL is almost universally performed under general anesthesia, with a double-lumen endobronchial tube in two consecutive sessions, with an interval of 1–2 weeks between sessions in approximately 50 % of centres. The use of saline warmed to 37 °C, drainage of lung lavage fluid by gravity and indications for WLL therapy in PAP were homogenous across centres.

There was great variation in the choice of the first lung to be lavaged: 50 % of centres based the choice on imaging, whereas 50 % always started with the left lung. The choice of position was also widely discordant; the supine position was chosen by 50 % of centres. Other aspects varied significantly among centres including contraindications, methods and timing of follow up, use of chest percussion, timing of extubation following WLL and lung isolation and lavage methods for small children. The amount of fluid used to perform the WLL is a critical aspect. Whilst a general consensus exists on the single aliquot of fluid for lavage (around 800 ml of warm saline, in adults) great variability exists in the total volume instilled per lung, ranging from 5 to 40 liters, with an average of 15.4 liters/lung.

**Conclusions:**

This international survey found that WLL is safe and effective as therapy for PAP. However these results also indicate that standardization of the procedure is required; the present survey represents the a first step toward building such a document.

**Electronic supplementary material:**

The online version of this article (doi:10.1186/s13023-016-0497-9) contains supplementary material, which is available to authorized users.

## Background

Whole lung lavage (WLL) is a therapeutic procedure [1] used to treat pulmonary alveolar proteinosis (PAP), a rare syndrome occurring in a heterogeneous group of lung diseases characterized by accumulation of lipoproteinaceous material in the alveoli, whicht impairs oxygen uptake and causes hypoxemic respiratory failure [2, 3]. Therapeutic efficacy derives from removal of the accumulated lipoproteinaceous material – primarily surfactant and necrotic cell debris – by physically ‘washing’ the alveoli with saline. WLL is usually performed under general anesthesia with lung separation obtained by a double-lumen endobronchial tube. While mechanical ventilation is maintained in one lung, the contralateral lung is repeatedly filled with saline and then drained by gravity. Typically, the lavage is accompanied by chest percussion to emulsify the surfactant sediment, and is continued until the lavage fluid becomes clear, usually judged by visual inspection.

Presently applied WLL procedures are based on the first description by Juan Ramirez Rivera in 1963 [1], but several centres have introduced modifications with the aim of improving the original method [4]. Although widely considered as the standard-of-care for autoimmune PAP [5], the WLL procedure, indications for its use, and the criteria to measure outcome have not been standardized among centres. Nor has the therapeutic effectiveness been compared for different secondary PAP syndromes. Further, centre-specific indications have never been compared, integrated to optimize or standardize WLL, or systematically disseminated. Moreover training on how to perform WLL involves an apprenticeship or in many cases is self taught; which obviously leads to additional variations among centres.

To begin laying the foundation for a consensus and experience-based, best-practice standardization of WLL, physicians performing WLL were surveyed by questionnaire on a global scale. Results concern the practice of WLL in adult and paediatric patients and include information on the procedure itself, local modifications, indications for use, contraindications, procedural monitoring, criteria to measure outcomes and complication rates.

## Methods

### WLL questionnaire development

This study was conducted as part of the e-RARE 2009 *EuPAPnet* study (http://www.alveolarproteinosis.eu/). An international ad hoc committee of physicians performing WLL and/or caring for PAP patients developed the survey questionnaire, which includes sections for physicians only performing WLL in either adult (≥18 years-old) or paediatric (<18 years-old) patients; only lobar or segmental bronchoscopic lavage (LSBL) in adult and/or paediatric patients; or both WLL and LSBL. The questionnaire is available in the Additional file 1.

### Data collection

The study cohort included physicians performing therapeutic lung lavage. Potential partecipants were identified either through a PubMed search (http://www.ncbi.nlm.nih.gov/pubmed) using the search terms ‘pulmonary proteinosis’, ‘alveolar proteinosis’, ‘alveolar phospholipidosis’, ‘lung lavage’, and ‘whole lung lavage’; or invited by an announcement placed in the European Respiratory Journal [6] or directly contacted: i.e., members of the international paediatric interstitial lung disease network; and acquaintances of steering committee members. Potential respondents were contacted by e-mail, interested individuals received the questionnaire by email, and all completed questionnaires that were returned were included in the analysis. All respondents gave written authorization for their responses to be included in the study.

### Analysis

Numeric variables were evaluated for normality with the Shapiro-Wilk test and expressed as the mean +/− SD or median +/− the interquartile range (IQR) as appropriate. Comparisons were made by one-way ANOVA with the Bonferroni correction. Categorical data were summarized numerically or expressed as a percentage and compared using the chi square or Fisher’s exact test as appropriate. Correlations among continuous variables were made using Pearson’s correlation coefficient. All comparisons were two-sided. P values of <0.05 were considered statistically significant. Analyses were performed using STATA, version 13.1 (Stata Corporation, College Station, Texas, USA). Three categories for the number of times WLL was conducted in a single patient (WLL < 2, ≥2 WLL <3, WLL >3) were utilized in analyzing results related to clinical outcomes.

Data were considered in a two-stage IPD meta-analysis, in order to correct the unavailability of single patient data. In the first stage, each individual study was analyzed as described in the meta-analysis protocol or analysis plan. As a second step, the results, or summary statistics, of each of these individual study analyses were combined to provide a pooled estimate of effect, as for a conventional systematic review.

## Results

Of the 79 centres identified worldwide that provide therapeutic lung lavage (52 centres in adults, 27 in children), 40 (50 %) expressed an interest in the study and were sent the questionnaire; of these 27 (33 %) completed and returned the documents (Table 1). Among these, seventeen centres treat only adults (14 only WLL, no one only SLBL, 3 both WLL and SLBL), seven treat only children (1 only WLL, 3 only SLBL, 3 both WLL and SLBL), and three treat both (2 only WLL, no one only SLBL, 1 both WLL and SLBL). Among centres providing both WLL and SLBL, WLL is considered the standard-of-care and SLBL treatment is reserved for patients judged unable to tolerate WLL. SLBL was also used in young children for whom double lumen endobronchial tubes of sufficiently small diameter are unavailable, and also to predict the therapeutic effectiveness of WLL in patients in whom this was uncertain, e.g., those with Niemann Pick disease.

**Table 1:** Centres participating in the survey

Centre	WLL	SLBL	Adult	Paediatric
Sheffield Children’s Hospital, UK	X	X		X
Royal Brompton Hospital, London, UK	X	X	X	X
Sheba Medical Centre, Tel-Hashomer, Tel Aviv U., Israel	X		X	X
Children’s Hospital Boston, USA	X			X
Kinderklinik und Kinderpoliklinik im, U. of Munich, Germany	X	X		X
Cincinnati Children’s Hospital, USA	X	X		X
Fondazione IRCCS Policlinico San Matteo, Pavia, Italy	X		X	
University of North Carolina at Chapel Hill, USA		X		X
Hacettepe University, Ankara, Turkey		X		X
U. Witwatersrand, Johannesburg, South Africa		X		X
Pavlov State Medical University, St. Petersburg, Russian Federation	X	X	X	
NHO Kinki-Chuo Chest Medical Centre, Osaka, Japan	X	X	X	
U. Medical College, Hangzhou, Zhejiang, China	X	X	X	
Lungenclinic Grosshandorf, Germany	X		X	
U. Hospital, Olomouc, Czech Republic	X		X	
Asklepios-Fachkliniken München Gauting, Germany	X		X	
Helsinki University Central Hospital, Finland	X		X	
Dept. of Respiratory Medicine, Cork University Hospital, Ireland	X		X	
Care Medicine Thoraxklinik, Heidelberg, Germany	X		X	
Serviço de Pneumologia- Hospital São João-Porto, Portugal	X		X	
Ruhrlandklinik-University of Duisburg Essen, Germany	X		X	
Pulmonary Division, University Hospital, Zurich, Switzerland	X		X	
Hopital Louis Pradel, Lyon, France	X		X	
Tokyo Medical U. Hachioji Medical Centre, Japan	X		X	
Kantonsspidal Aarau, Switzerland	X		X	
St. Antonius Hospital Nieuwegein, The Netherlands	X		X	
Kempten-Oberallgäu Hospital, Immenstadt, Germany	X		X	X

1. WLL in Adult Patients

**Fig. 1:**
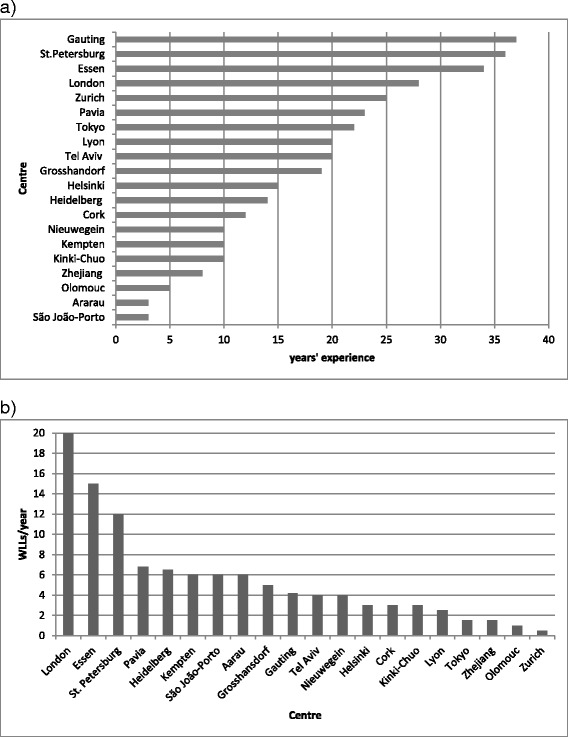
**a**. Years’ experience of each centre in performing WLL in adult PAP patients. **b**. Mean number of WLLs performed per centre annually, in adult PAP patients

Twenty centres reported performing WLL in adults (Table 1). The mean (+/− SD) duration of experience was 18 +/− 11 years and varied substantially among centres (Fig. 1a). The mean number of WLL procedures performed annually per centre was 5.61 +/− 5.04 and also varied among centres (Fig. 1b).

### Indications and contraindications for WLL therapy

Indications for WLL varied among centres (Table 2). Specific indications included an unspecified decline in lung function, a decline in resting PaO_2_, worsening of lung disease severity judged radiographically based on a comparison of serial chest computed tomograms or chest radiograms (using visual assessment in all except two centres that use semi-quantitative criteria), decline in diffusing carbon monoxide capacity (DLCO), decline in forced vital capacity (FVC), decline in resting oxygen saturation by pulse oximetry (SpO_2_) or an increase in respiratory symptoms.

**Table 2:** Indications for WLL therapy

Indications for WLL	% of centres
Unspecified decline in lung function	100
Decline in resting PaO_2_	90
Chest X-ray or CT	79
Decline in DLCO	70
Decline in FVC	63
Decline in SpO2	58
Symptoms	42
Other	15

Three centres also reported using WLL for indications other than PAP, including: accidental inhalation of activated charcoal, alveolar hemorrhage, silicoproteinosis, lipoid pneumonia, silicosis or cryptogenic fibrosing alveolitis (CFA).

Contraindications to WLL were applied by six centres and included severe cardiovascular disease, heart failure, sepsis, significant lung infection and end-stage pulmonary fibrosis.

### Interval between treatment of right and left lungs

Most (17/20) centres performed WLL in separate sessions on each lung for a given patient. The time separating WLL therapy for each lung was 2.9 ± 1.18 weeks and varied from 24 h at one centre to 6–12 weeks at another centre. Only one centre routinely treated both lungs in one session, while two other centres treated lungs in one or two sequential sessions depending on patient tolerance.

### Number of WLL treatments Per patient required in autoimmune PAP

**Fig. 2:**
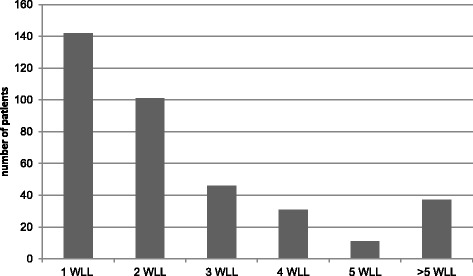
Stratification of adult autoimmune PAP patients according to the number of WLL procedures received

Of the approximate 368 PAP patients for whom data was evaluated, the number of WLL procedures received by PAP patients during the follow-up period was 2.5 +/− 1.5 in five years. Of these patients, two thirds required one lavage, and only 10 % required more than five WLL procedures (Fig. 2). The interval between consecutive bilateral WLL procedures was 8 +/− 6.45 months but varied from 1 week to several years.

Because the clinical course of autoimmune PAP is variable, we evaluated whether the experience of the centre (number of years in activity and number of WLL procedures performed annually) and the volumes used for WLL might relate to clinical outcome. We found no correlation in these parameters (*χ*^2^ = 0.1238 and 0.4905, respectively) suggesting that neither the experience level or volume of WLL fluid used were significantly correlated with clinical outcome.

### Complications in WLL

Based on an estimated 1110 WLL procedures, the centres reported specific complication rates ranging from a median of 16 % for transient fever (the most common complication) to 0.8 % for pneumothorax (Table 3).

**Table 3:** Complications in WLL

Complication	Frequency (%)^a, b^
Fever	18.0
Fluid leakage	4.0
Hypoxemia	14.2
Wheezing	6.1
Pneumonia	5.0
Headache	0
Respiratory acidosis	0
Transient neuropathy	0
Pleural effusion	3.1
Prolonged mechanical ventilation	0
Metabolic acidosis	0
Pulmonary thromboembolism	0
Pneumothorax	0.8
Transient cardiac ischemia	0
Cardiac arrest	1.1^c^

^a^Based on an estimated total of 1110 WLLs. Estimation of the total number of WLLs is the sum of the total per centre calculated as: median WLL number per year multiplied by the number of years’ experience

^b^The frequency of complications was estimated as the mean value of frequencies reported by the centres

^c^Cardiac arrest occurred only in 1 case out of 5 WLL reported, only at 1 centre

### Follow up after lung lavage therapy

All centres reported performing a short-term assessment (chest x-ray and functional assessment) after WLL over a period that ranged from 2 h to 2 weeks. Some (9/20) centres also performed a follow up chest computed tomogram (CT) scan at a variable time ranging from 7 days to 6 months. All centres reported performing medium- to long-term follow up using conventional radiological and functional parameters including chest CT scan (12/20 centres – routinely at 8, and on an ‘as indicated basis’ at 4 months). Six centres performed follow up biomarker evaluations including serum LDH, GM-CSF autoantibody levels and less commonly SPA, SPD, KL-6, CEA, Cyfra 21–1. Two centres followed up WLL procedures by using a quality of life questionnaire.

### Implementation of the WLL procedure

#### Lung isolation

The lungs must be isolated during WLL so that one can be lavaged while the other is ventilated to maintain the necessary gas exchange. This is usually achieved by positioning a double-lumen endobronchial tube, which ensure an adequate seal to prevent fluid spillage from the treated lung into the ventilated lung. A left-sided double-lumen endobronchial tube is the most common choice in adults and larger children. The minimum size of the double-lumen tube is 26 Fr.

#### Anesthesia

At all centres, WLL is performed under general anesthesia (Additional file 2: Table S1), typically by intravenous anesthesia, most commonly with a combination of propofol, an opioid and a neuro-muscular blocking agent. Two centres use volatile anesthetic agents. At about half the centres (9/20), a procedure referred to as ‘lung atelectasis/degassing’ is performed prior to WLL. Patient parameters typically monitored during WLL are indicated in Table 4.

**Table 4:** Parameters monitored during anesthesia/WLL

Parameter monitored	N° of centres (%)
EKG	20 (100 %)
SaO_2_	18 (90 %)
Non invasive blood pressure	13 (65 %)
End tital CO_2_	13 (65 %)
Arterial catheter blood pressure	11 (55 %)
CVC	7 (35 %)
Bispectral index	3 (15 %)
Blood gas analysis	3 (15 %)
Pulmonary compliance	1 (5 %)

#### Lung selection

The method used to decide which lung to treat first varied among centres. About half (12/20) used radiographic information to identify and treat the more severely affected lung first, which was based on visual inspection of the chest CT (9 centres), chest x-ray (2 centres), or a combination of chest x-ray and CT (1 centre). At six centres, the decision was independent of lung-specific severity: the left lung was always treated first, due to its smaller relative size. At about half of the centres (9/20), a procedure referred to as ‘lung atelectasis/degassing’ is performed prior to starting the lavage of each lung. Degassing of one lung is obtained by ventilation with 100 % oxygen followed by forced lung deflation with negative airway pressure and subsequent airway opening occlusion maintained for 10 to 15 min up to absorption atelectasis of the whole lung. Lung degassing is intended to help the lavage fluid reach the alveoli more easily and evenly.

#### Patient position

The position of the patient during WLL varied considerably among centres: 12 (60 %) centres utilized a supine position; 6 (30 %) centres utilized a full lateral position at 90°; 2 centres utilized a moderate lateral position at 30° to 45° inclination. Of those utilizing a lateral position, all but one centre ventilated the non-dependent lung and lavaged the dependent lung. Only one centre uses a combination of full lateral position, dependent lung ventilation and non-dependent lung lavage, having observed better oxygenation due to better ventilation/perfusion matching, no problems with lavage spillover and more convenient access of the lavaged lung for manual chest percussion. Moreover, 7 centres indicated that patient positioning included the Trendelenburg position to improve lavage recovery and increase alveolar clearance.

#### Lavage fluid and administration

All Centres used saline warmed to 37 °C for WLL. Supplements used at some centres included (number of centres using the supplement): N-acetyl cysteine (1), aminophylline (1), hydrocortisone (1), sodium bicarbonate (2) and gaseous oxygen (1). One centre modified the filling-drainage cycle by adding manual ventilation of the lavaged lung half-way through the draining time, intended to improve the removal of material from the lung [7]. All centres infused saline by gravity. One centre used a specified hydrostatic head of 30 cm relative to the mid-thorax.

**Fig. 3:**
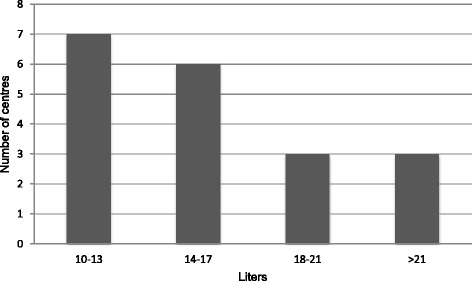
Stratification of total amount of fluid (litres) used to lavage a single lung

The volume of saline aliquots infused during repeated, sequential lung lavage varied greatly, ranging from 80 (SLBL) to 1,650 ml (WLL), with an average of 800 ± 331 ml. The total lavage volume also varied widely among centres, ranging from 5 to 40 litres with most (13/20) centres using less than 18 litres per lung (Fig. 3). The mean total volume among the 20 centres was 15.4 +/− 6.8 litres per lung. Only one centre regularly utilized a fixed volume (12 litres per lung).

#### Chest percussion

Most (14/20) centres used chest percussion to emulsify the PAP sediment (~90 % lipid) in order to improve therapeutic efficiency. However, the method and timing varied greatly. Ten (50 %) centres utilized manual chest percussion and four (20 %) centres used mechanical percussion. The timing of chest percussion also varied with some centres utilizing it during the entirety of each infusion-drainage cycle, some during the drainage portion only, some only after filling.

#### Duration and termination

The duration of WLL for one lung varied from 2 to 6 h with an average of 6 +/− 1 h for both lungs. All centres terminated WLL when the drained fluid became markedly less cloudy than observed at the start of therapy; this was usually based on visual inspection of the gross appearance of the recovered fluid. Two centres also used an objective measure of protein content.

#### Recovery period

Mechanical ventilation of both lungs in the intensive care unit (ICU) was maintained from 20 min to 19 h after completion of WLL, with an average of 5 +/− 1.5 h. Most patients were discharged from the ICU within 5 h after extubation.

2. Lung Lavage in Paediatric Patients

**Fig. 4:**
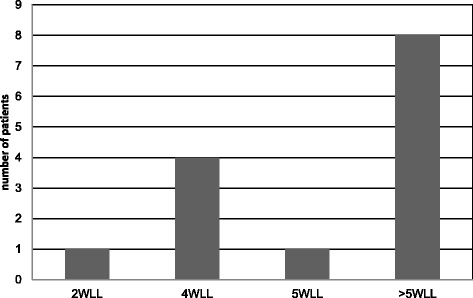
Stratification of paediatric PAP patients according to the number of procedures received

Seven centres reported performing lung lavage in paediatric patients (4 - WLL or SLBL, 3 only WLL). Three additional centres perform lung lavage only by bronchoscopy (Table 1). The mean (+/− SEM) duration of experience was 16 +/− 17 years, but this varied among centres (not shown). The mean number of SLBL procedures performed annually per centre was 5.75 +/− 5.83 and also varied among centres (not shown). The average number of WLL treatments per paediatric PAP patient (2.3 ± 1.52) was similar to that of adults (2.5 ± 1.48). Indicators for pediatric WLL were also similar to those for adults. The interval between WLL treatment for each lung was on 5 +/− 2.8 days. All centres used warm saline without additives. The volume of saline infused ranged from 250 to 500 ml per aliquot. One centre infused saline under a hydrostatic pressure of 30–40 cm H_2_O. The total volume applied per lung varied from 4 to 14 litres. Three centres applied chest percussion, two of them manually and one mechanically either during the filling or drainage periods. The average duration of WLL in children was 3.5 h. Smaller patients were weaned from mechanical ventilation over a period of between 2 and 48 h with a mean of 12.7 +/− 23.5 h. Follow up evaluation after WLL was similar to that of adult PAP patients except that CT scans were not routinely used. In contrast to adults, paediatric PAP patients received a greater number of lavage procedures (Fig. 4), possibly because of differing disease (i.e., GM-CSFRa deficiency instead of autoimmune PAP) or the more frequent use of SLBL, which treats only a segment or lobe, rather than WLL, which treats the entire lung. At one centre, the intubation method for children, as well as the decision to perform WLL or lobar/segmental lavage via bronchoscopy, is based on patient size. In children large enough to possibly tolerate a double lumen tube, single-lumen endotracheal tubes of increasing diameter are first ‘test-fitted’ to identify the maximum possible size while avoiding subglottic trauma. Intubation is then performed with a double-lumen tube of the size identified. For children too small for a double-lumen tube, intubation of the lung to be lavaged is achieved with a single-lumen endotracheal tube, while ventilation of the other lung is achieved by ventilating the trachea around the endotracheal tube. The most frequent complications of WLL in children are reported in Table 5.

**Table 5:** Complications of WLL in children

Complication	Overall occurrence rate
Hypoxemia	13 %
Fluid leakage	6 %
Pleural effusion	6 %
Fever	5 %
Wheezing	3 %
Pneumonia	3 %
Pneumothorax	1 %

## Discussion

As a first step in developing an evidence-based, best-practice approach to standardizing WLL therapy in PAP, we made the results of a global survey of physicians performing WLL in adults and children. Respondents included 20 centres in 14 countries performing WLL in adults and 10 centres in 6 countries performing WLL in paediatric patients. Some aspects of WLL were similar among centres, including the method for selecting the first lung to be treated, treatment of each lung at different sessions separated by several days to weeks, use of general anesthesia, a double-lumen endotracheal tube (in adults), saline warmed to 37 °C, and drainage of lung lavage fluid by gravity. Other aspects of the procedure varied significantly among centres, including indications for treatment, contraindications, methods and timing of the follow up evaluation, patient position during WLL, the volume of saline infused, use of chest percussion, timing of extubation after WLL and lung isolation and lavage methods for small children. The length of experience among centres varied widely, but the overall rate of serious, procedure-related complications was low. Limited number of diseases requiring WLL therapy, procedural variations and their relationship to differences in outcomes among patients and centres, all support the need for integrated data from multiple centres in order to develop a best-practices approach to standardizing WLL therapy.

Important findings of our WLL survey include: in spite of its clear therapeutic usefulness, it is not available at most medical centres; it is frequently taught by informal apprenticeship or is even self-taught and it varies among centres with respect to the procedure itself, indications for use, and assessment of benefit. We found 161 reports on the use of WLL therapy for PAP in the NCBI PubMed database including several technical descriptions [8–12]. There was a consensus on indications for WLL therapy in PAP, which included worsening of lung function/gas exchange (100 % of centres), followed by radiographic evidence of deterioration (79 %), and then symptomatic worsening (42 % of centres). Notwithstanding this consensus, the parameter used to determine each varied considerably among centres. Even though the majority of centres used ca. 800 ml of warmed saline for each single infusion during WLL, centres varied in their use and choice of additives, and the total volume of saline used to treat each lung. These differences have important implications for the conduct of clinical trials evaluating new therapeutic approaches.

Interestingly, only 65 % of the centres employed an observation period before performing the WLL. With the exception of rapidly progressive cases, this would be advisable as spontaneous improvement is possible. Even though, complete spontaneous remission, previously believed to occur in up to 30 % of cases [13], is now known not to exceed 10 % of cases [14, 15].

The absence of an association between variability in the clinical course among PAP patients (indicated by the different number of WLL treatments needed to obtain remission) and differences WLL practice among centres (volume of saline used, number of procedures performed annually, length of experience performing WLL) suggest that intrinsic disease characteristics predominate over technical differences and experience in applying WLL among centres. On the other hand, the low number of complications and absence of a correlation with experience status may be related to the fact that the centres filling out the questionnaire were all relatively experienced. In fact, several authors stated that they treated patients who were sent for a tertiary referral after a complicated WLL procedure in an inexperienced hospital.

Although WLL is an invasive procedure, it has been determined to be safe and associated with a low rate of procedure-related morbidity (~18 %, including, in order of decreasing frequency, fever, spillage of saline into the ventilated lung, and worsened hypoxemia). SLBL [16] is an alternative to WLL and is regarded as less invasive, but neither its therapeutic effectiveness, nor its rate of complications have been adequately studied and thus was not considered in the present study.

This study has some limitations, which interfere with the statistical analysis of the results, notably: the incomplete participation of physicians/centres who are known to practice WLL therapy. Thus, our results may not adequately reflect the practice of WLL at all centres or, potentially, important improvements made at centres not surveyed. Notwithstanding these limitations, these results provide the first useful data for developing consensus documents regarding technical implementation, indications, and evaluation of WLL therapy. For this reason, the questionnaire has been included in an Additional file 1 for use by other centres wishing to provide additional data regarding their centre’s practice of WLL. Even though few centres performing WLL in children were identified/included, one important problem identified with this survey was the lack of availability of double lumen endotracheal tubes of sufficiently small size for use in small children.

## Conclusions

Our study (as well as the literature) provides data for the development of a consensus document and experience-based, best-practice standardization of WLL. We conclude that the assembly of an international task force on the conduct of WLL therapy in adults and children would be helpful in this regard.

## Abbreviations

CEA, carcinoembryonic antigen; CFA, cryptogenic fibrosing alveolitis; CT, computed tomogram; Cyfra 21–1, cytokeratin 19 fragment; DLCO, carbon monoxide diffusing capacity; FVC, forced vital capacity; GM-CSF, granulocyte-macrophage colony-stimulating factor; ICU, intensive care unit; IQR, interquartile range; KL-6, Krebs von den Lungen 6; LDH, lactate dehydrogenase; LSBL, lobar or segmental bronchoscopic lavage; NCBI, National Center for Biotechnology Information; PAP, pulmonary alveolar proteinosis; SPA, surfactant protein A; SPD, surfactant protein D; SpO_2_, resting oxygen saturation; WLL, whole lung lavage

## Additional files

Additional file 1: Therapeutic lavage for pulmonary alveolar proteinosis. A questionnaire for an international survey. (DOCX 126 kb) Additional file 2: Pharmacologic approaches utilized to support WLL. (DOCX 14 kb)
