# Supplementary material for: Whole lung lavage therapy for pulmonary alveolar proteinosis: a global survey of current practices and procedures
Source: Orphanet J Rare Dis. 2016 Aug 31;11(1):115. doi: 10.1186/s13023-016-0497-9 (PMC5006612; doi:10.1186/s13023-016-0497-9)
Supplement: Additional file 1: — Therapeutic lavage for pulmonary alveolar proteinosis. A questionnaire for an international survey. (DOCX 126 kb) [file 13023_2016_497_MOESM1_ESM.docx]

**THERAPEUTIC LAVAGE FOR PULMONARY ALVEOLAR PROTEINOSIS**

**A QUESTIONNAIRE FOR AN INTERNATIONAL SURVEY**

**Instructions**

**Please include data referring to the last five years on average**

**If you perform both bronchoscopic lobar/segmental lavage and whole lung lavage, please complete all three sections.**

**If you perform bronchoscopic segmental lavage, please fill the first section only**

**If you perform only whole lung lavage, please skip to the third section.**

**Please answer to questions yes (Y) or (N), if not otherwise specified.**

**Fill in written responses where indicated.**

**Please complete the form in english**

**First section : for those who perform bronchoscopy lobar/segmental lavage**

**Pediatric patients Adult patients Both**

1. Why have you chosen to perform bronchial/segmental lavage to manage PAP patients? Please explain briefly

|  |
| --- |

1. Since when you perform bronchial/segmental lavage?

|  |
| --- |

1. How many PAP patients do you see on yearly basis?

(average)

|  |
| --- |

1. How many patients do you submit to lavage on yearly basis?

(average)

|  |
| --- |

1. How many lavages do you perform on yearly basis?

(average)

|  |
| --- |

1. What is the average number of lavages for individual PAP patient in your series during the last five years?

|  |
| --- |

1. What are the indications for initiating lavage in your patients?
   1. Deteriorating lung function

Based on worsening of:

FVC

DlCO

PaO2 Δ from baseline

SaO2 Δ from baseline

exercise induced drop in PaO2 Δ from baseline

exercise-induced drop in Sat Δ from baseline

If not in respiratory failure, how many % point change over baseline

Method? 6MWT

Treadmill

Worsening of Δ AaO2

at rest exercise

Δ from baseline

7.2 Worsening of Chest Rx/CT scan

Do you use an objective tool to evaluate the severity on imaging (specify)?

No Yes (if yes, please specify below)

7.3 Worsening patient symptoms

Based on dyspnea?

Based on another?

7.4 Do you observe before treatment?

If yes, how many months?

7.5 Other indications (Iist below)?

|  |
| --- |

1. Anesthesia.

8.1 Do you perform lavage under local or general

anesthesia?

8.2 If you perform the treatment under general anesthesia, please describe briefly technical issues

|  |
| --- |

8.3 Please list the parameters checked during the lavage

| ECG ? |  |
| --- | --- |
| SatO2 ? |  |
| Non invasive blood pressure ? |  |
| Artery catheter for blood pressure ? |  |
| Others (please specify) |  |

1. Do you use flexible or rigid bronchoscopy?
2. How many segments/lobes do you lavage in a single session? Comments?

|  |
| --- |

1. How do you select the lobe to be lavaged?

|  |
| --- |

1. What is the position of the patient during the lavage?

Seated supine

12.1 Do you perform chest percussion during the lavage?

If yes, please describe briefly the method of percussion used

Manual Mechanical

1. Fluid

13.1 Do you use cold or warm (37°C) saline?

13.2 Do you add anything to the saline (specify)?

|  |
| --- |

13.3 How many saline milliliters are in a single injected aliquots in adult patients?

in pediatric patients?

13.4 Do you calculate the recovery after single injected aliquots?

Yes/no if yes, what is the average %?

13.5 Do you take into account the negative balance to decide when stop the lavage?

Yes/no

13.6 Do you use a fixed or varying total amount of fluid?

How much (in average, if varying)?

|  |
| --- |

13.7 If you use varying amount of fluid, is your decision to stop the lavage based on what?

|  |
| --- |

14. How long is the procedure?

15min 30min 45min 60min

more than 60min

15. Short term efficacy of the lavage (within first week)

15.1 Do you check the effects of the lavage after the procedure?

15.2 If yes, which parameters do you check?

15.2.1. Chest X Ray

How far from the procedure?

15.2.2 Functional parameter(s)? What?

|  |
| --- |

How far from the procedure?

15.2.3 Lung CT Scan?

How far from the procedure?

16. Further segmental/lobar lavages.

16.1 Since it is unlikely that a single segmental/lobar lavage is enough to improve the PAP lung function/imaging, several lavages are necessary to obtain an improvement. In your protocol, have you a fixed number of lavages or they are performed based on the outcome of the previous ones ??

|  |
| --- |

16.2 Which is the average interval between two consecutive lavages?

|  |
| --- |

17. Long term follow up

Which parameters do you check in the follow up of the PAP patient ? Please state also at what interval from each other.

17.1 Chest X ray

|  |
| --- |

17.2 Functional parameters? What?

|  |
| --- |

17.3 Lung CT Scan?

|  |
| --- |

17.4 Any biomarkers (including GM-CSF autoantibodies)?

|  |
| --- |

18. Complications

Please list the complications occurred in your lavaging experience, stating the average prevalence

| Complication | % |
| --- | --- |
| Fever |  |
| Pneumonia |  |
| Hypoxemia |  |
| Pleural effusion |  |
| Bronchospastic reaction |  |
| Pneumothorax |  |
| Other (please specific) |  |
|  |  |

19. Contraindications

Beside the usual contraindications to bronchoscopy, did in your experience happen specific conditions that precluded the lavage to be performed?

|  |
| --- |

20. Please include below any comments you would like to raise

|  |
| --- |

**Second section: for those who perform both lobar/segmental lavage and whole lung lavage**

1. On what basis you select PAP patients for one option or the other? Is there any objective evaluation tool you use? Please specify.

|  |
| --- |

**Third section : for those who perform whole lung lavage**

**Pediatric patients Adult patients Both**

1. Why did you start to perform whole lung lavage to manage PAP patients? Please explain briefly

|  |
| --- |

1. How long have you been performing whole lung lavage?

|  |
| --- |

1. How many PAP patients do you see on yearly basis?

(average)

|  |
| --- |

1. How many patients do you submit to whole lung lavage on yearly basis?

(average)

|  |
| --- |

1. How many whole lung lavages do you perform on yearly basis?

(average)

|  |
| --- |

1. What is the average number of lavages for individual PAP patient in your series during the last five years?

|  |
| --- |

7. What are the indications for initiating lavage in your patients?

- 1. Deteriorating lung function

Based on worsening of:

FVC

DlCO

PaO2 Δ from baseline

SaO2 Δ from baseline

exercise induced drop in PaO2 Δ from baseline

exercise-induced drop in Sat Δ from baseline

If not in respiratory failure, how many % point change over baseline

Method? 6MWT

Treadmill

Worsening of Δ AaO2

at rest exercise

degree

7.2 Worsening in Chest Rx or lung CT scan

7.2.1 Do you use an objective tool to evaluate the severity on imaging (specify)?

|  |
| --- |

7.3 If there is no worsening in lung function or imaging, do you consider the patient request of lavaging, for subjective feeling of reduced performances?

Yes/no

7.4 Do you consider an observation period before performing lavage (for possible spontaneous improvement?)

Yes/no if yes, how many months?

8. Anesthesia.

8.1 Please describe briefly the technique used (induction and maintenance)

|  |
| --- |

8.2 Do you perform atelectasis/degasing pre WLL? Yes/no

8.3 Please describe the parameters checked during the lavage

| ECG ? |  |
| --- | --- |
| SatO2 ? |  |
| Non invasive blood pressure ? |  |
| Artery catheter for blood pressure ? |  |
| CVC catheter ? |  |
| ET CO2 ? |  |
| Others (please specify) |  |

9. Kind of procedure

9.1 Do you generally perform whole lung lavage of both lungs in a single session?

9.2 Do you generally perform whole lung lavage of single lung in a single session?

9.2.1 If yes, what is the average interval between the two lavages?

|  |
| --- |

10. How do you select the lung to be lavaged first?

|  |
| --- |

11. Position and technique

11.1 What is the position of the patient during the lavage?

|  |
| --- |

11.2 Do you use specific maneuvers/modifications of the position during the lavage?

|  |
| --- |

11.3 Others? comments?

|  |
| --- |

12. Fluid

12.1 Do you use cold or warm (37°C) saline?

12.2 Do you add anything to the saline?

|  |
| --- |

12.3 How many saline milliliters are injected a single aliquot?

in adult patients

in pediatric patients

12.4 Please describe briefly the infusion/recovery technique

|  |
| --- |

12.5 Do you use a fixed or varying amount of fluid?

How much liters in total per lung (in average, if varying)?

|  |
| --- |

12.6 If you use varying amount of fluid, is your decision to stop the lavage based on what?

|  |
| --- |

13. Percussion

13.1 Do you perform chest percussion during the lavage?

13.2 If yes, please describe briefly the method of percussion used

Manual Mechanical

13.3 At what stage of the lavage do you perform the percussion?

|  |
| --- |

14. How long does the procedure last ?

14.1 Single lung lavage.including anesthesia

2 -4 hours 4-6 hours more than 6 hours

14.2 Double lung lavage including anesthesia

4-6 hours 6-8 hours more than 8 hours

14.3 How long after the end of the lavage do you start the weaning from mechanical ventilation ?

|  |
| --- |

14.4 If the lavage is performed in the ICU, when is the patient transferred to a non-ICU bed?

<2-4 2-4 4-6 >6 hours

15. Short term efficacy of the lavage

15.1 Do you check the effects of the lavage after the procedure?

15.2 If yes, which parameters do you check?

15.2.1. Chest X Ray

How far from the procedure?

15.2.2 Functional parameter(s)?

What?

|  |
| --- |

How far from the procedure?

15.2.3 Lung CT Scan?

How far from the procedure?

16. Long term follow up

Which parameters do you check in the follow up of the PAP patient? Please state also at what interval from each other

16.1 Chest X ray

|  |
| --- |

16.2 Functional parameters? What?

|  |
| --- |

16.3 QoL questionnaire?

|  |
| --- |

16.4 Lung CT Scan?

|  |
| --- |

16.5 Any biomarkers (including GM-CSF autoantibodies)?

|  |
| --- |

17 Multiple whole lung lavages

17.1 How do you decide that the PAP patient would need another whole lung lavage? Do you use the same parameters as described at point 7?

17.2 If not, what?

|  |
| --- |

17.3 Which is the average interval between two consecutive lavages?

|  |
| --- |

17.4 What percentage of your PAP series requires more than one lavage? Please list ranking according to number of lavages ?rs

| **Number of WLL** | **Number of patients** | **%** |
| --- | --- | --- |
| 1 |  |  |
| 2 |  |  |
| 3 |  |  |
| 4 |  |  |
| 5 |  |  |
| More than 5 |  |  |

18. Complications

Please list the complications occurred in your lavaging experience, stating the average prevalence

| Complication | % |
| --- | --- |
| Fever |  |
| Pneumonia |  |
| Hypoxemia |  |
| Pleural effusion |  |
| Bronchospastic reaction |  |
| Pneumothorax |  |
| Cardiac arrest |  |
| Leakage of fluid (airway.pleural space) |  |
| Others (please specify) |  |

19. Have you ever excluded a candidate to whole lung lavage? In your experience, what are the contraindications?

|  |
| --- |

20. Have you ever performed whole lung lavage with the help of particular equipment, such as extracorporeal circulation, hyperbaric chamber, ECMO, etc?

If yes, please state rationale, kind and number

|  |
| --- |

21. Have you ever performed whole lung lavage in a clinical setting other than proteinosis? If yes, please specify kind and number of procedures

|  |
| --- |

22. Please include below any comments you would like to raise

|  |
| --- |
