# Supplementary material for: Whole lung lavage therapy for pulmonary alveolar proteinosis: a global survey of current practices and procedures
Source: Orphanet J Rare Dis. 2016 Aug 31;11(1):115. doi: 10.1186/s13023-016-0497-9 (PMC5006612; doi:10.1186/s13023-016-0497-9)
Supplement: Additional file 2: — Pharmacologic approaches utilized to support WLL. (DOCX 14 kb) [file 13023_2016_497_MOESM2_ESM.docx]

| **Centre** | **Induction** | | | **Maintenance** | | | **Atelectasis/degasing** |
| --- | --- | --- | --- | --- | --- | --- | --- |
|  | **opioid** | **hypnotic** | **relaxation** | **opioid** | **hypnotic** | **relaxation** |  |
| **Brompton** | remifentanil | propofol | vecuronium | remifentanil | propofol | vecuronium | Yes |
| **China** | ND | ND | ND | ND | ND | ND | No |
| **Cork** | ND | ND | ND | ND | ND | ND | No |
| **Essen** | remifentanil | propofol | cisatracurium | remifentanil | propofol | cisatracurium | Yes |
| **Ararau** | ND | propofol | Yes | ND | propofol | Yes | Yes |
| **Gauting** | Yes | propofol | yes | Yes | propofol | yes | Yes |
| **Grosshandorf** | ND | ND | ND | ND | ND | ND | No |
| **Heidelberg** | ND | propofol | Yes | ND | propofol | ND | ND |
| **Helsinki** | fentanyl | propofol | rocuronium | fentanyl | propofol | rocuronium | No |
| **Immenstadt** | fentanyl | propofol | ND | fentanyl | propofol | ND | No |
| **Lyon** | ND | ND | Yes | ND | ND | Yes | No |
| **Nieuwengein** | fentanyl | propofol | atracurium | fentanyl | propofol | atracurium | Yes |
| **Olomuc** | ND | ND | ND | ND | ND | ND | No |
| **Osaka** | ND | thiopental | ND | remifentanil | sevoflurane | ND | Yes |
| **Pavia** | fentanyl | propofol | vecuronium | fentanyl | propofol | vecuronium | Yes |
| **Porto** | remifentanil | propofol | rocuronium | remifentanil | propofol, sevoflurane | rocuronium | Yes |
| **St.Petersburg** | ND | ND | Yes | ND | ND | Yes | No |
| **Tel Aviv** | ND | ND | Yes | ND | isoflurane | Yes | Yes |
| **Tokio** | fentanyl | propofol | ND | fentanyl | propofol | ND | No |
| **Zurick** | ND | ND | ND | ND | ND | ND | Yes |

**Supplemental material** Supplemental Table 1
